# Topographical Landmarks for the Identification of Branches of Mandibular Nerve and Its Surgical Implications: A Cadaveric Study

**DOI:** 10.7759/cureus.20120

**Published:** 2021-12-02

**Authors:** Ariyanachi Kaliappan, Vidhya Meena S, Sivakumar Manivasagam, Vanangamudi Kaliappan, Lakshmi Jyothi

**Affiliations:** 1 Anatomy, All India Institute of Medical Sciences, Bibinagar, Hyderabad, IND; 2 Anatomy, Thiruvallur Medical College, Chennai, IND; 3 Anatomy, Thiruvannamalai Medical College, Thiruvannamalai, IND; 4 Orthopaedics, Government Theni Medical College, Theni, IND; 5 Microbiology, All India Institute of Medical Sciences, Bibinagar, Hyderabad, IND

**Keywords:** accessory mandibular foramen, mandibular foramen, inferior alveolar nerve, lingual nerve, massetric nerve

## Abstract

Introduction

Basic knowledge of anatomy is crucial in providing predictable, safe, and efficacious mandibular anesthesia as the mandibular nerve is vulnerable to injury during dental procedures and other surgical manoeuvers. The lack of availability of the appropriate topographical bony landmarks for the location of the branches of this nerve often accounts for iatrogenic injuries and the failure to obtain adequate local anesthaesia. Hence we aimed to describe the topographical landmarks of the branches of the mandibular nerve and their variations in the infratemporal fossa.

Methodology

In 16 formalin-fixed cadavers, irrespective of the sex of the cadavers, bilateral dissection of the infratemporal fossa was done after identifying the necessary bony landmarks. The mandibular nerve and its branches were traced out and the required measurements were taken using the digital vernier caliper. The results were statistically analysed for mean, range, and standard deviation.

Results

The masseteric nerve is 15.87+/-1.64 mm superior to the lowest point on the mandibular notch. The lingual nerve in the third molar area is at the depth of 24.75+/-2.38 mm from the angle of the mandible (gonion), making an angle of 50° with the base of the mandible. 20.13+/-3.1 mm inferior to the mandibular notch is the precise location of the mandibular foramen which allows access to the inferior alveolar nerve. The incidence of accessory mandibular foramen in the dissected samples is 9.37%.

Conclusion

The topography of the masseteric nerve, lingual nerve, and inferior alveolar nerve was studied using constant and reliable bony landmarks in the cadaver which might aid effective dental and facio-maxillary surgical procedures. However, the outcome of this study could not be applied to paediatric patients as the subjects were restricted to adult cadavers.

## Introduction

The largest division of the trigeminal nerve is the mandibular nerve. Mandibular nerve block analgesia which involves the temporary blockage of the lingual nerve, inferior alveolar nerve, and the masseteric nerve is normally a safe and rewarding method employed in the management of pain during dental, oral, and maxillofacial surgical procedures. But a few patients do encounter the undesirable side effects of the impairment of neurosensory function after mandibular block anaesthesia. The masseteric nerve block technique is being used for the reduction of dislocated mandibular condyles and for facial reconstruction techniques [1]. Nevertheless, because the facial parameters on which certain surgical methods are based often vary, it is not always feasible to detect the nerve. Variable anatomical localisation of the lingual nerve ensures proper understanding of its position which will reduce the possible damage and morbidity [2,3]. Further, the failure rate of inferior alveolar nerve block procedure is about 20-25% as there is a lack of ideal bony landmarks to localize the mandibular foramen. The failure of anaesthesia caused by the inferior alveolar nerve block might be explained by a lack of proper understanding of the anatomical placement of the mandibular foramen, especially as the location of the mandibular foramen varies from person to person [4]. This study was carried out to gain adequate knowledge of the topography of these branches in the cadavers which could be reproduced in the patients during the surgical procedures involving these nerves.

## Materials and methods

**Figure 1:**
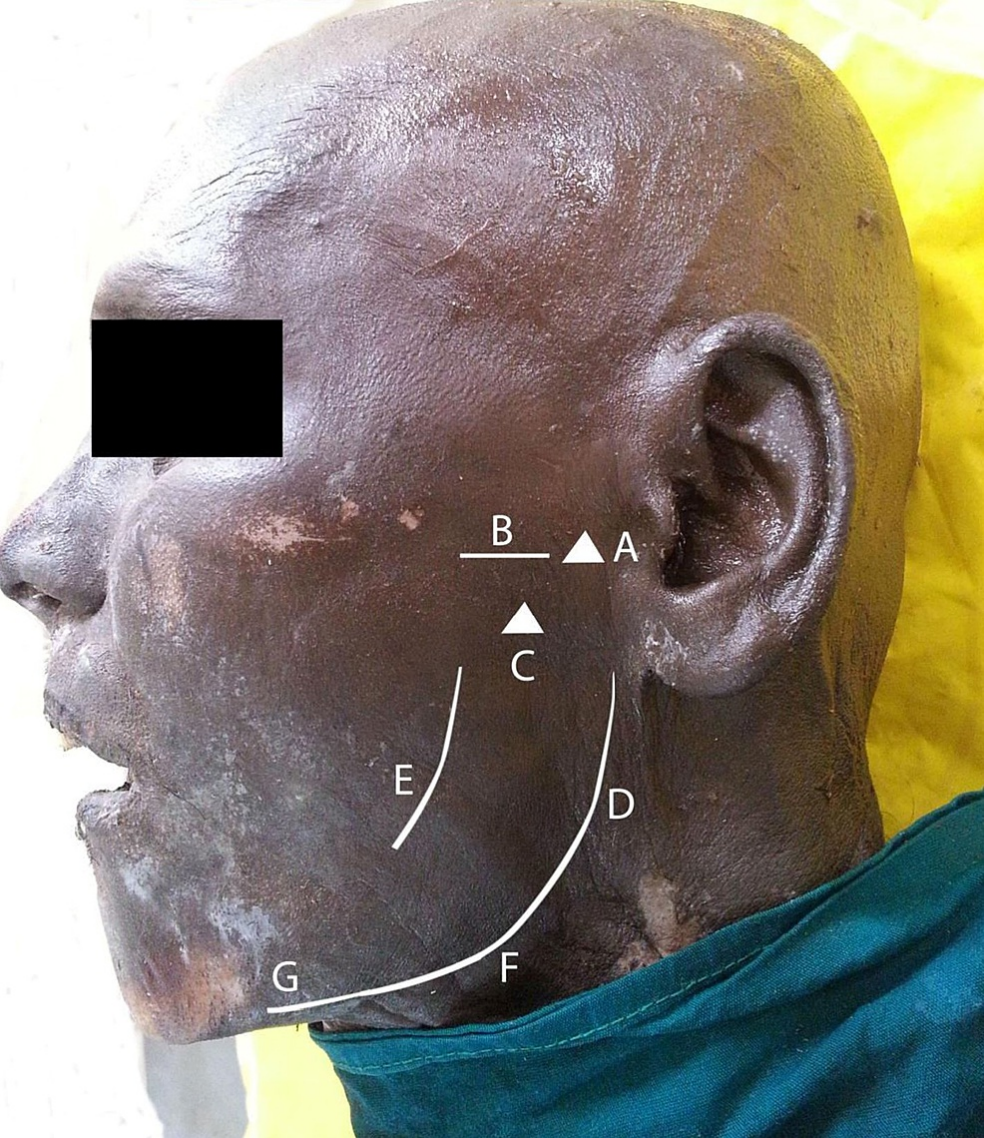
External palpable bony landmarks related to the infratemporal fossa A – TM joint (temporomandibular joint); B – zygomatic arch; C – mandibular notch/sigmoid notch; D – posterior border of the ramus of the mandible; E – anterior border of the ramus of the mandible; F – gonion/angle of the mandible; G – base of the mandible

The study was approved by the Post Graduate Research Monitoring Committee (Reg. No: PGRMC/ANAT/02/2014) and Institutional Ethics Committee (Project No: JIP/IEC/SC/2014/8/600) and was carried out in the Department of Anatomy. Sixteen formalin-fixed cadavers fit to be dissected in the infratemporal fossa were included for the study. The cadaver number, age, sex, and date of dissection were recorded. The palpable bony landmarks of the infratemporal fossa - the angle of the mandible (gonion), the base of the mandible, anterior border of the ramus of the mandible, posterior border of the ramus of the mandible, temporomandibular joint, zygomatic arch, and mandibular notch - were identified (Figure 1).

**Figure 2:**
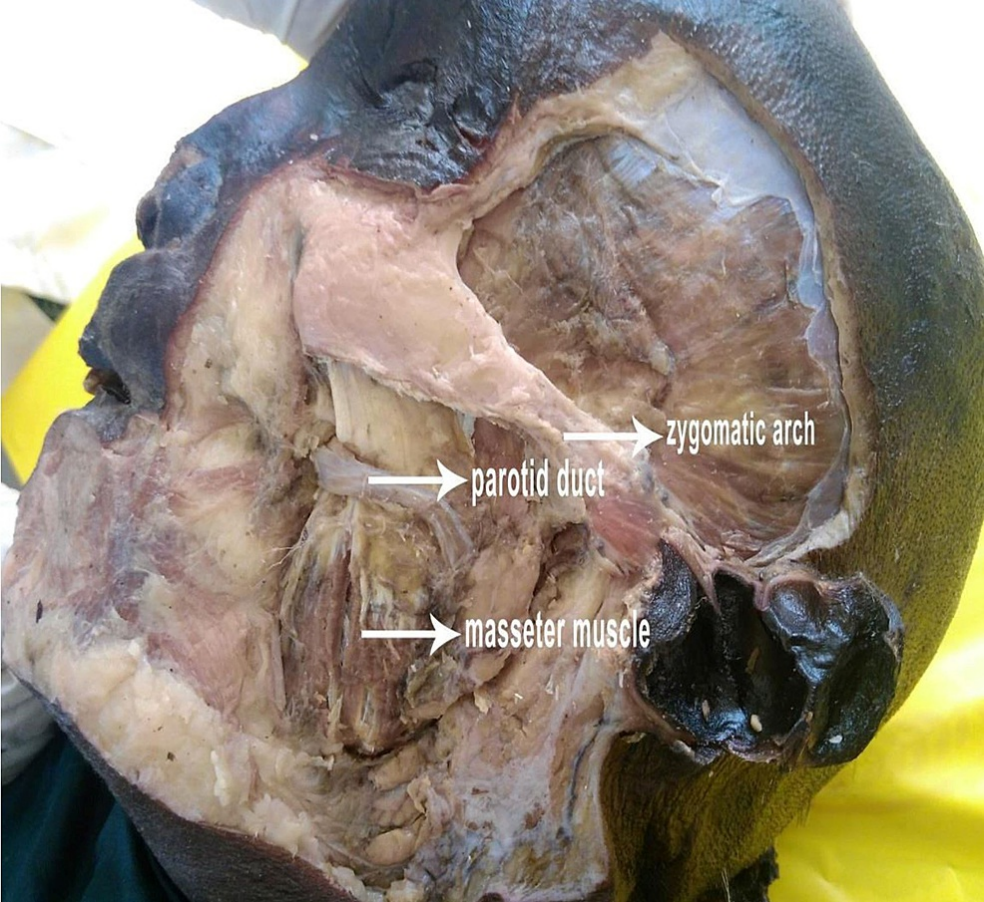
Exposure of masseter muscle

**Figure 3:**
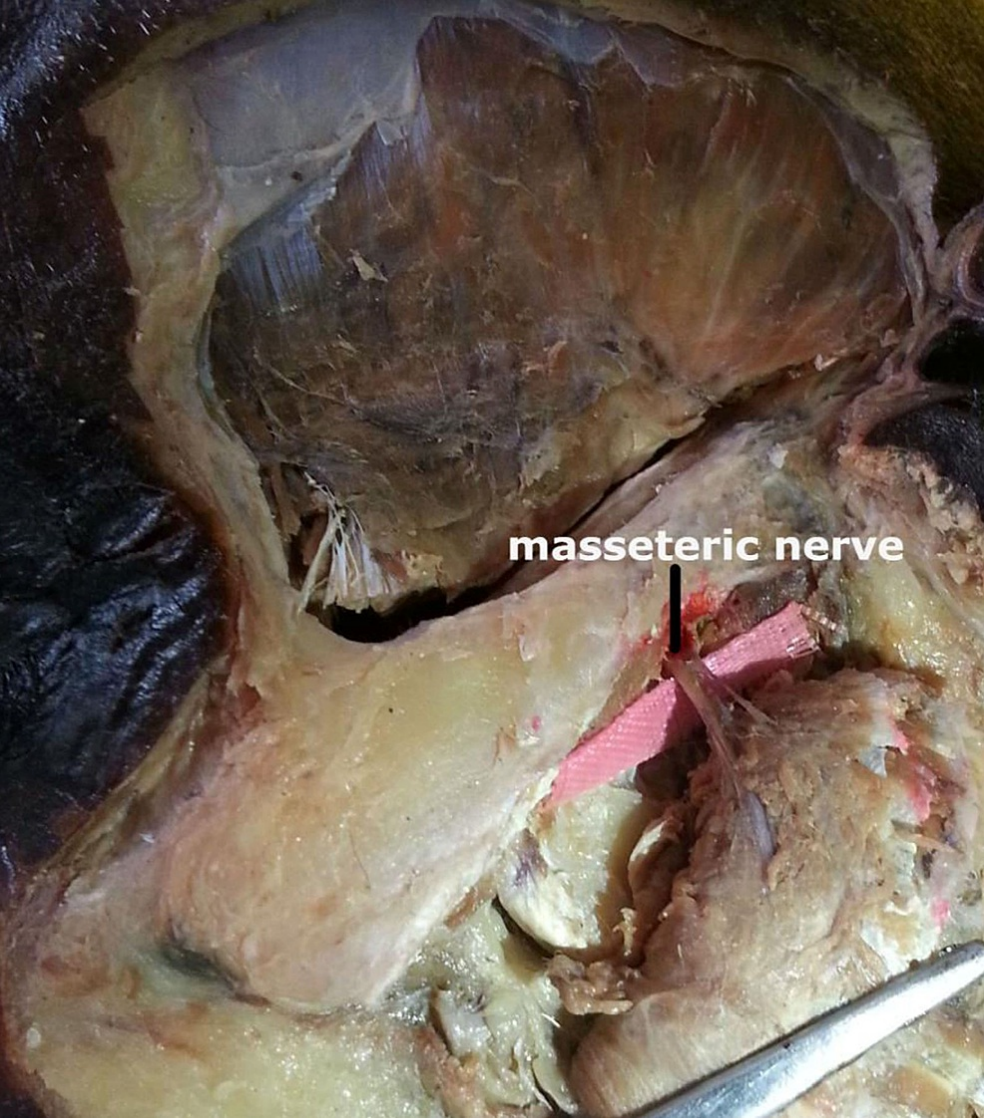
Exposure of masseteric nerve

An incision was made on the skin over the zygomatic arch and along the anterior border of the ramus of the mandible. After removing the skin and the fascia, an incision was made gently over the exposed masseter muscle along the lower border of the zygomatic arch. Care was taken not to damage the underlying masseteric nerve. The muscle belly was then gently reflected downwards and laterally, exposing the masseteric nerve. It was noted that the masseteric nerve enters the masseter from the undersurface of the muscle after exiting via the mandibular notch, also called the sigmoid notch (Figures 2, 3).

**Figure 4:**
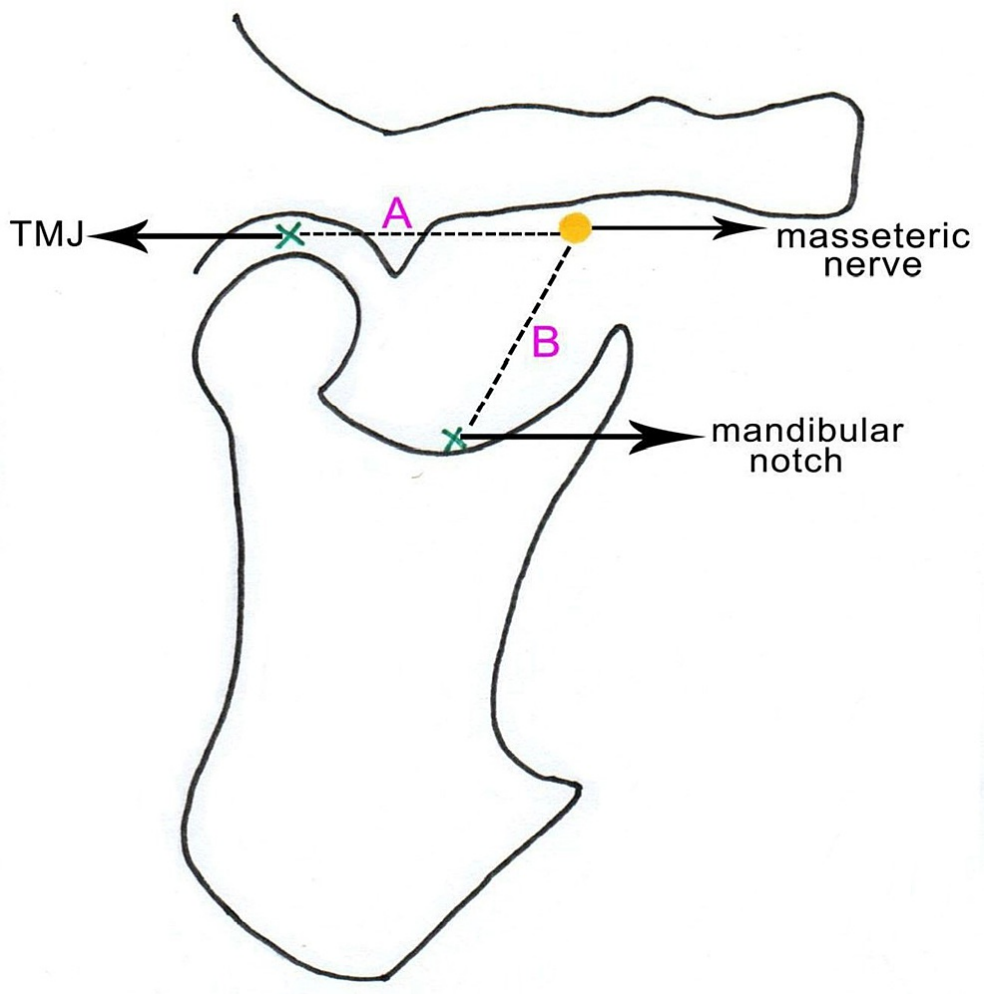
Topography of the masseteric nerve A – Distance from the mid-point of the temporomandibular joint capsule to the masseteric nerve (immediately below the lower border of the zygomatic arch) B – Distance from the lowest point on the mandibular notch to the masseteric nerve TMJ - temporomandibular Joint

The distance between the masseteric nerve and the midpoint of the temporomandibular joint capsule was measured with the help of a digital vernier caliper. The distance between the exit of the nerve immediately below the zygomatic arch and the lowest point of the mandibular notch was also measured using the vernier caliper (Figure 4).

**Figure 5:**
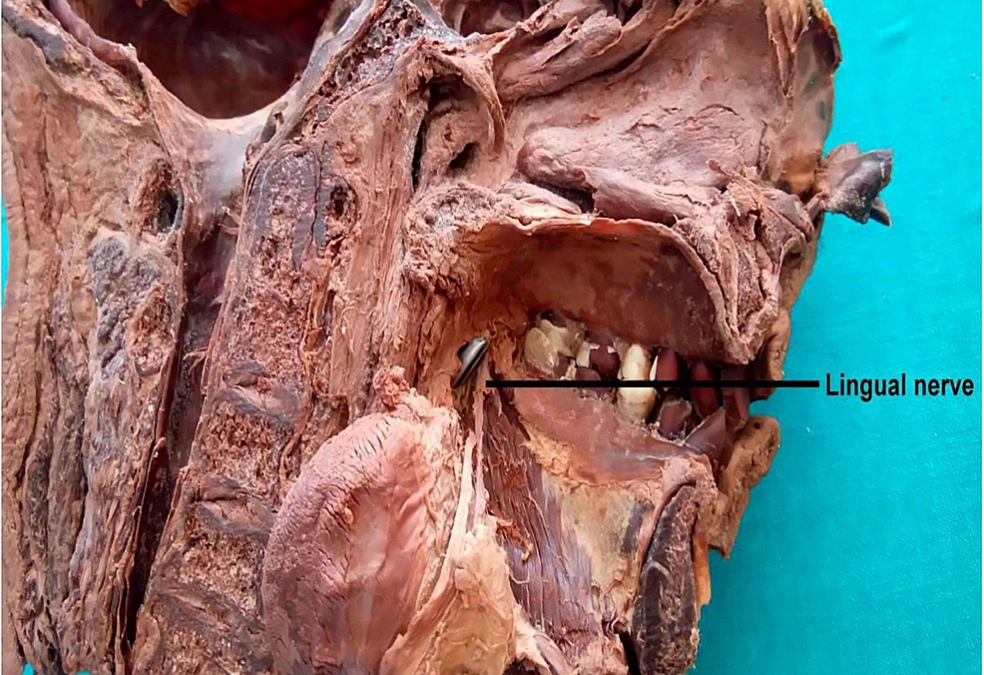
Exposure of lingual nerve in the third molar area

Then with the help of a cross-cut hand saw, the mandible was cut into two halves at the symphysis menti. Each half of the bone was then disarticulated from the corresponding temporomandibular joint. The zygomatic arch was broken followed by meticulous dissection and removal of temporalis, medial pterygoid, and lateral pterygoid muscles. The mandibular nerve with its trunk, anterior division, and posterior division was identified. The bifurcation spot of the lingual nerve and inferior alveolar nerve from the posterior division was noted. Otic ganglion was traced and found medial to the mandibular nerve trunk under the foramen ovale. The posterior division of the mandibular nerve and its branches were traced. Any variations or abnormal communications between the branches if present were noted. The tongue was pushed aside and an incision was made in the mucosa around the lingual plate of the third molar area. The lingual nerve was traced. A probe was inserted at the gonion at an angle of 50° (50 degrees) with the base of the mandible to reach the lingual nerve in the third molar area (Figure 5).

**Figure 6:**
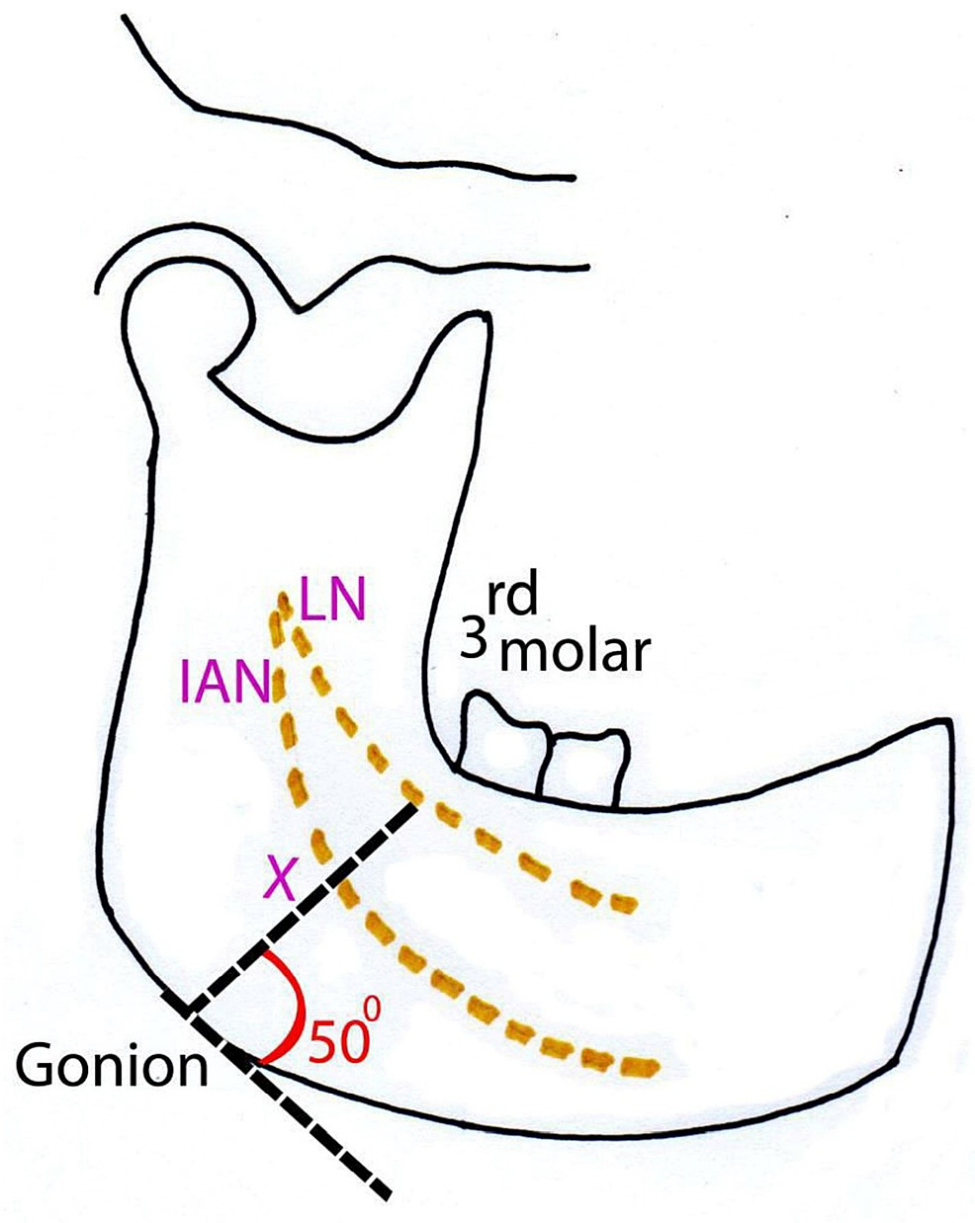
Lingual nerve topography - lateral view X – Distance from the gonion (angle of the mandible) to the lingual nerve in the third molar area at an angle of 50° with the base of the mandible LN - lingual nerve; IAN - inferior alveolar nerve

Then the distance from the point of entry of the probe at the gonion to the lingual nerve was measured (Figure 6).

**Figure 7:**
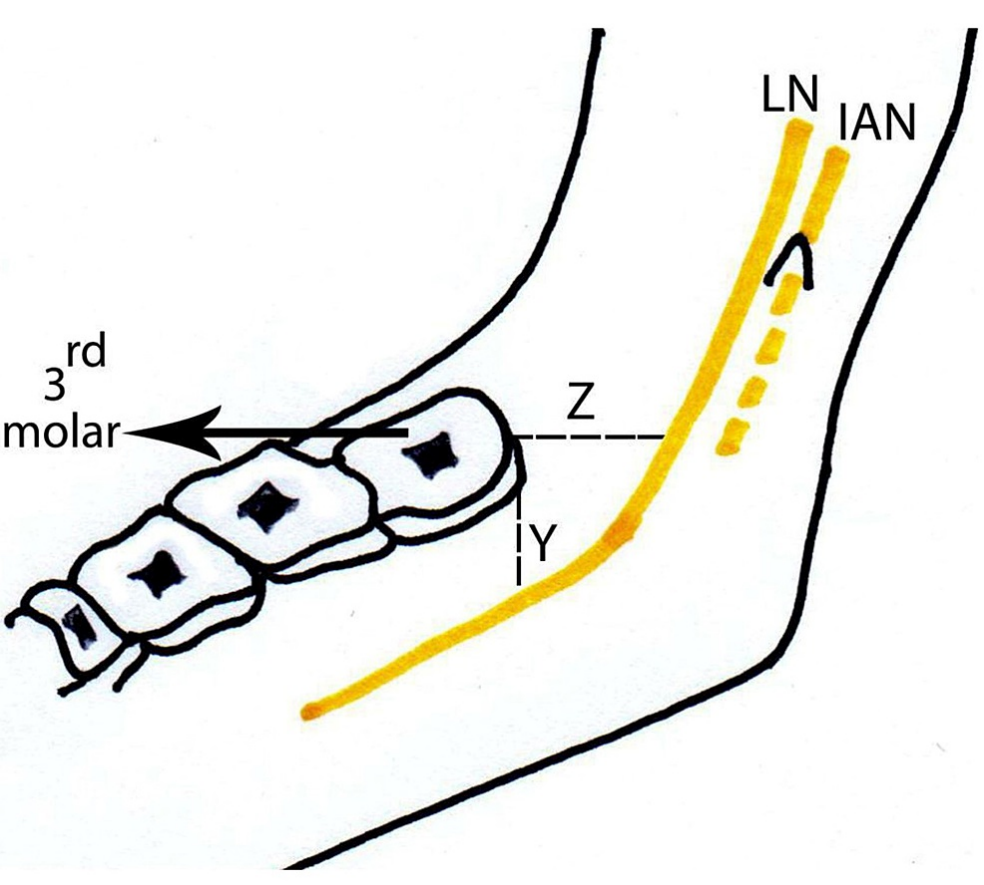
Lingual nerve topography - medial view Y – Distance from the lingual nerve to the alveolar crest of the (distolingual area of) third molar Z – Distance from the lingual nerve to the lingual plate of the (distolingual area of) third molar LN - lingual nerve; IAN - inferior alveolar nerve

The vertical distance from the lingual nerve to the alveolar crest of the distolingual area of the third molar and the horizontal distance from the lingual nerve to the lingual plate of the distolingual area of the third molar were also measured with the digital vernier caliper (Figure 7).

**Figure 8:**
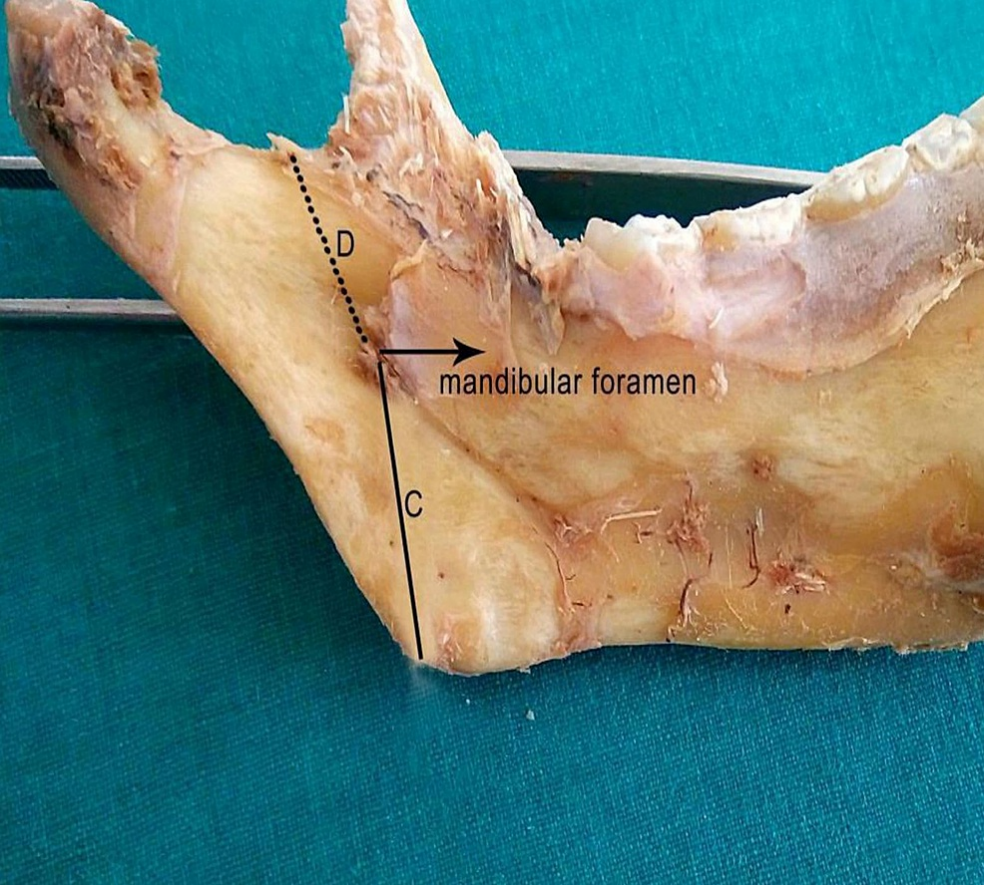
Topography of mandibular foramen C – Distance from the mandibular foramen to the angle of the mandible, at an angle of 50° with the lower border of the base of the mandible D – Distance from the mandibular foramen to the lowest point (center) of the mandibular notch

**Figure 9:**
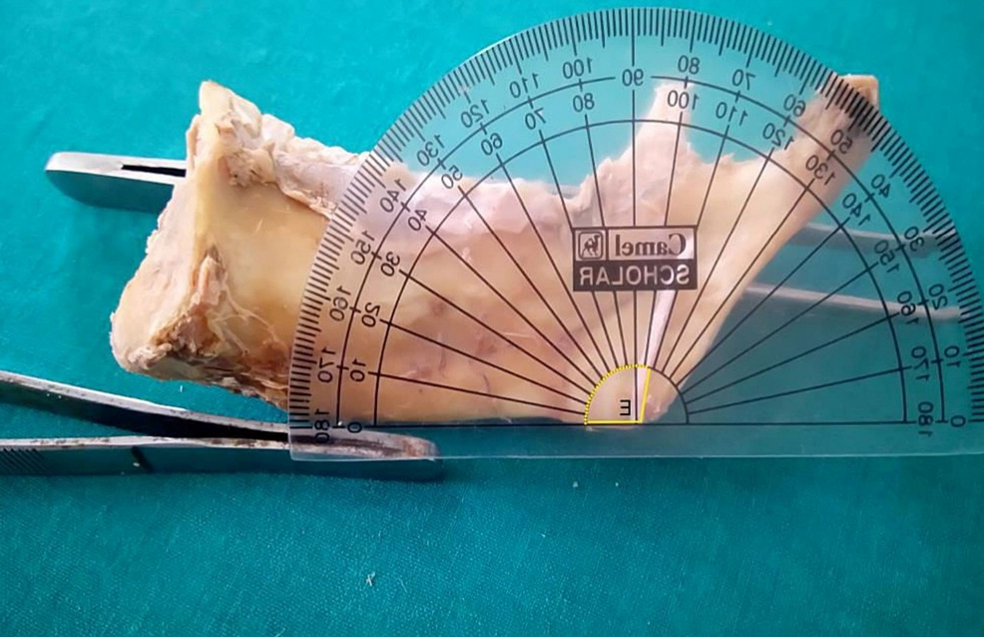
Topography of the mandibular foramen E – Angle formed by the mandibular foramen at the angle of the mandible (gonion) with the lower border of the base of the mandible

The mandibles were removed from the cadavers; then all the muscle fibres attached to them were removed. The mandibular foramen's precise location was investigated (Figures 8, 9).

**Figure 10:**
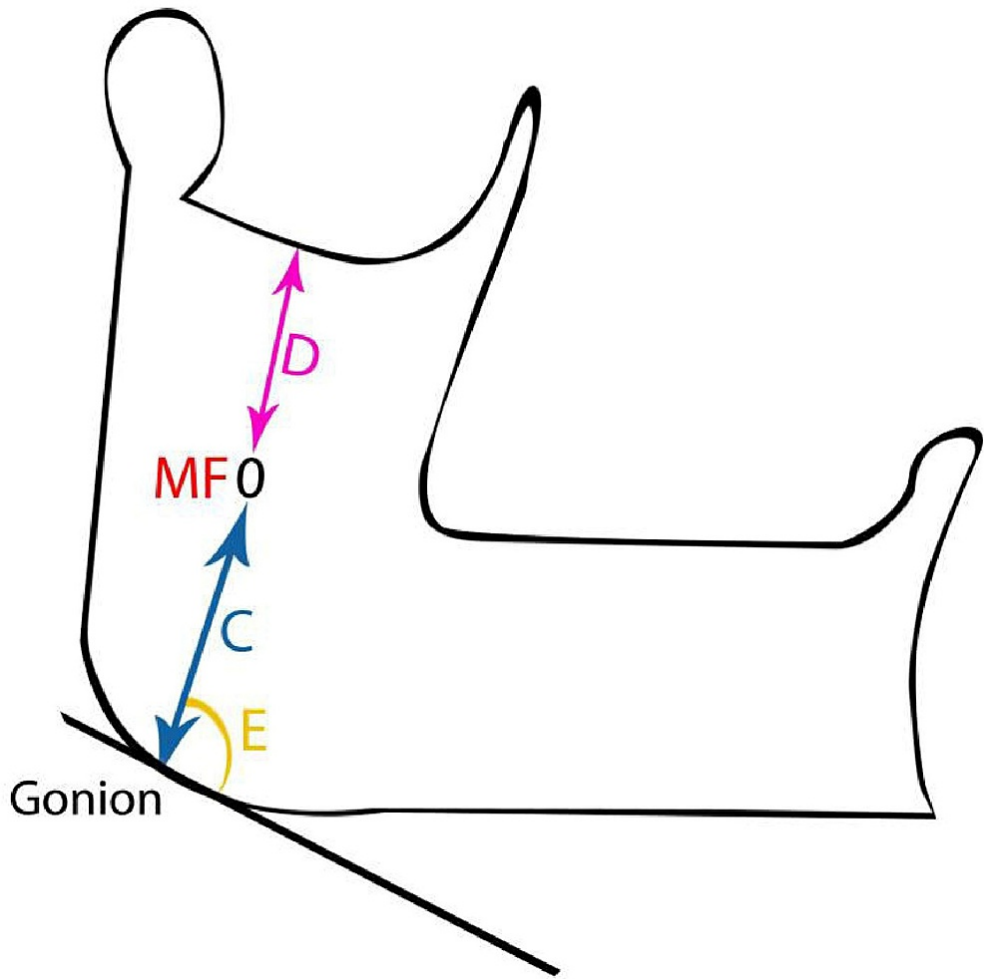
Landmarks for the mandibular foramen topography C – Distance from the mandibular foramen to the angle of the mandible, at an angle of 50° with the lower border of the base of the mandible D – Distance from the mandibular foramen to the lowest point (center) of the mandibular notch E – Angle formed by the mandibular foramen at the angle of the mandible (gonion) with the lower border of the base of the mandible MF - mandibular foramen

The vernier calliper was used to calculate the distance between the gonion (angle of the mandible) and the mandibular foramen. The distance between the lowest point (middle of the mandibular notch) on the sigmoid notch and the mandibular foramen was also measured. The angle formed between the mandibular foramen (at the angle of the mandible) and the base of the mandible was measured using a protractor (Figure 10). 

**Figure 11:**
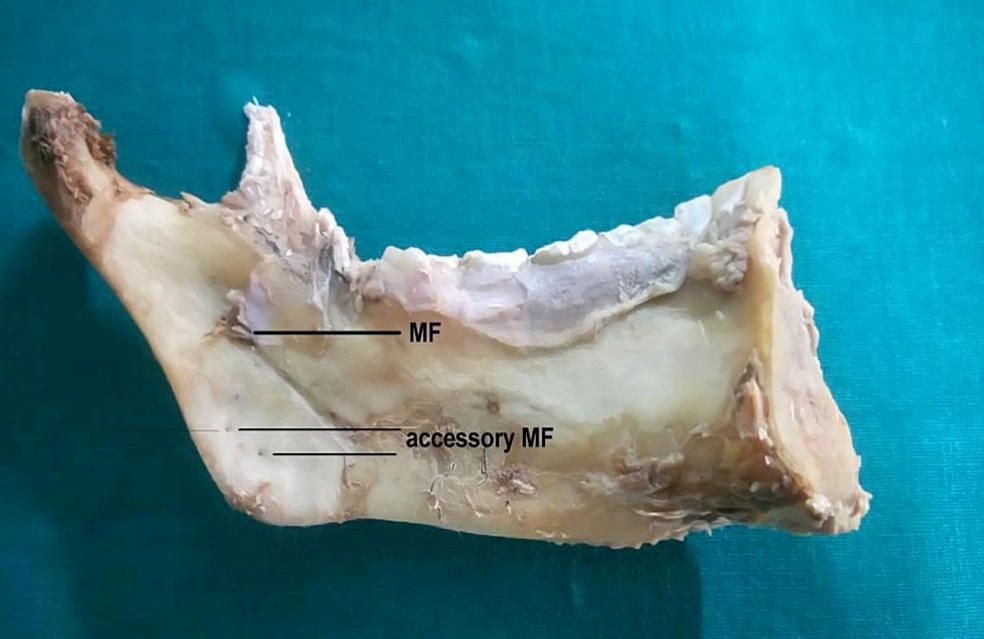
Accessory mandibular foramen MF - mandibular foramen

After that, the mandibles were checked for any accessory mandibular foramen (Figure 11).

GraphPad Instat 3 (San Diego, USA) was used to accomplish the statistical analysis. For every parameter, descriptive statistics were calculated. The Student's t-test was used to compare each parameter on the right and left sides.

## Results

Out of 16 formalin-fixed cadavers dissected, 14 (87.5%) were males and 2 (12.5%) were females (Table 1).

**Table 1:** Sex distribution of the cadavers

Sex of the cadavers	No. of cadavers	No. of mandibular nerves traced
Male	14	28
Female	2	4
Total	16	32

Masseteric nerve topography

The topographical landmarks for the masseteric nerve were studied in relation to the temporomandibular joint and mandibular notch (Table 2).

**Table 2:** Masseteric nerve topography in relation to temporomandibular joint and mandibular notch A – Distance from the mid point of the temporomandibular joint capsule to the masseteric nerve (immediately below the lower border of zygomatic arch) B – Distance from the lowest point on the mandibular notch to the masseteric nerve The masseteric nerve was 16.15 ± 2.32 mm medial to the midpoint of the temporomandibular joint capsule immediately below the lower border of the zygomatic arch. The masseteric nerve was 15.87 ± 1.64 mm superior to the lowest point on the mandibular notch.

Measurement	Mean ± SD (mm) n = 32	Range (mm)
A	16.15 ± 2.32	12.5 – 21.5
B	15.87 ± 1.64	13 - 18

The measurements related to the topography of the masseteric nerve were compared between the right and the left sides (Table 3).

**Table 3:** Bilateral comparison of the measurements related to the topography of masseteric nerve A – Distance from the mid-point of the temporomandibular joint capsule to the masseteric nerve (immediately below the lower border of the zygomatic arch) B – Distance from the lowest point on the mandibular notch to the masseteric nerve In the 16 formalin-fixed cadavers dissected, no statistically significant difference was observed in the measurements related to the topography of the masseteric nerve between the right and the left side (p>0.05)

Measurement	Side	Mean ± SD (mm) n = 16	Range (mm)	P-value
A	Right	16.25 ±2.29	12.5-21.5	0.823
	Left	16.06 ± 2.40	13-21	
B	Right	15.93 ±1.61	13.5-17.5	0.833
	Left	15.81 ±1.72	13-18	

Lingual nerve topography

The topographical landmarks for the lingual nerve were studied in relation to the gonion, alveolar crest of the distolingual area of the third molar, and the lingual plate of the distolingual area of the third molar (Table 4).

**Table 4:** Lingual nerve topography in relation to the gonion, alveolar crest and lingual plate of the third molar X – Distance from the gonion to the lingual nerve in the third molar area at an angle of 50° with the base of the mandible Y – Distance from the lingual nerve to the alveolar crest of the distolingual area of the third molar Z – Distance from the lingual nerve to the lingual plate of the distolingual area of the third molar The lingual nerve in the third molar area was at the depth of 24.75 ± 2.38 mm from the gonion at an angle of 50° with the base of the mandible. The distance from the lingual nerve to the alveolar crest of the third molar was 6.49 ± 0.58 mm. The distance from the lingual nerve to the lingual plate of the third molar was 2.55 ± 0.55 mm.

Measurement	Mean ± SD (mm) n=32	Range (mm)
X	24.75 ±2.38	22-30
Y	6.49 ± 0.58	5.4-7.5
Z	2.55± 0.55	1.7-4.0

The measurements related to the topography of the masseteric nerve were compared between the right and the left sides (Table 5)

**Table 5:** Bilateral comparison of measurements related to topography of lingual nerve X – Distance from the gonion to the lingual nerve in the third molar area at an angle of 50° with the base of the mandible Y – Distance from the lingual nerve to the alveolar crest of the distolingual area of the third molar Z – Distance from the lingual nerve to the lingual plate of the distolingual area of the third molar In the 16 formalin-fixed cadavers dissected, no statistically significant difference was observed in the measurements related to the topography of the lingual nerve between the right and the left sides (p>0.05).

Measurement	SIDE	Mean ± SD (mm) n=16	Range (mm)	p-value
X	Right	24.75± 2.32	22-30	0.999
	Left	24.75 ± 2.52	22-30	
Y	Right	6.54 ± 0.56	5.4-7.4	0.634
	Left	6.44 ± 0.55	5.4-7.5	
Z	Right	2.56 ± 0.67	1.7-4	0.916
	Left	2.54 ± 0.42	1.7-3	

Mandibular foramen (for inferior alveolar nerve) topography

The topographical landmarks for the mandibular foramen (for inferior alveolar nerve) were studied in relation to the gonion (angle of the mandible), the lowest point (centre) of the mandibular notch, and the angle formed by the mandibular foramen with the base of the mandible (Table 6) 

**Table 6:** Mandibular foramen topography in relation to the angle of the mandible and the mandibular notch C – Distance from the mandibular foramen to the angle of the mandible, at an angle of 50° with the lower border of the base of the mandible D – Distance from the mandibular foramen to the lowest point (centre) of the mandibular notch to the mandibular foramen E – Angle formed by the mandibular foramen at the angle of the mandible with the base of the mandible The mandibular foramen was 21 ± 3.33 mm superior to the gonion at an angle of 98° ± 5° with the base of the mandible. The mandibular foramen was 20.13 ± 3.1 mm inferior to the mandibular notch.

Measurement	Mean ± SD n=32	Range (mm)
C (mm)	21 ± 3.33	13-27
D (mm)	20.13 ± 3.1	14-25
E(degree)	98 ± 5	85 -110

The measurements related to the topography of the masseteric nerve were compared between the right and the left sides (Table 7).

**Table 7:** Bilateral comparison of measurements related to the topography of mandibular foramen C – Distance from the mandibular foramen to the angle of the mandible, at an angle of 50° with the lower border of the base of the mandible D – Distance from the mandibular foramen to the lowest point (centre) of the mandibular notch to the mandibular foramen E – Angle formed by the mandibular foramen at the angle of the mandible with the base of the mandible In the 16 formalin-fixed cadavers dissected, no statistically significant difference was observed in the measurements related to the topography of the mandibular foramen between the right and the left sides (p>0.05)

Measurement	Side	Mean ± SD n=16	Range	p-value
C (mm)	Right	21.18 ± 3.12	15-26	0.755
	Left	20.81 ± 3.61	13-27	
D (mm)	Right	20 ± 3.48	14-25	0.824
	Left	20.25 ± 2.79	16-24	
E (degree)	Right	98.43 ± 5.97	85-110	0.739
	Left	97.8 ± 4.46	90-110	

Accessory mandibular foramen

Accessory mandibular foramen was observed in 9.37% of the specimens dissected (Figure 11).

## Discussion

The masseteric nerve block is a novel technique used in the management of the pain and spasm of the masseter muscle caused by the dislocated mandibular condyle [5-11]. Recently the masseter muscle is being used for facial reanimation [12]. However, there is a paucity of knowledge regarding the precise topography of the masseteric nerve. In the current study, only the constant bony landmarks - the temporomandibular joint, zygomatic arch, and mandibular notch were taken into consideration to frame the topography of the masseteric nerve. This is in contrast to the previous studies where the soft tissue landmarks were included to identify the nerve. Collar et al., suggested a subzygomatic triangle that included the zygomatic arch, temporomandibular joint, and frontal branch of the facial nerve for the localisation of the masseteric nerve [13]. In the present study, in all the cadavers dissected, masseteric nerve emerged out of the mandibular notch immediately below the lower border of the zygomatic arch. This is in accordance with the results of Cotrufu et al., who demonstrated the nerve immediately below the zygomatic arch to run within the muscle belly [14]. However, this contradicts the observation by Borschel et al., where the nerve emerged out of the mandibular notch 1 cm inferior to the zygomatic arch [12]. Kaya et al. also suggested the exit of the nerve through the mandibular notch 13.8±4.5 mm inferior to the zygomatic arch [15]. In our observation, the masseteric nerve was 16.15 ± 2.31 mm medial to the midpoint of the temporomandibular joint capsule immediately below the lower border of the zygomatic arch and 15.87 ± 1.64 mm superior to the lowest point of the mandibular notch, whereas Kaya et al., proposed that the masseteric nerve was related 10.6±2.7 mm medial to the temporomandibular (TM) joint and 7.8±2.00 mm above the mandibular notch [16]. In this current study, there was no significant statistical disparity in the parameters between the right and the left side implying bilateral symmetry of the masseteric nerve.

The lingual nerve is more susceptible to injury during oral surgical procedures such as third molar extraction, implant placements, mandibular osteotomies, and mandibular fracture repair [16,17]. Studies have concluded that the anatomical localisation of the lingual nerve is highly variable which has resulted in an increased incidence of lingual nerve damage. In this study, an attempt was made to localise the lingual nerve in relation to the third molar and observed that the mean distance from the lingual nerve to the alveolar crest of the third molar was 6.49 ± 0.58 mm and the mean distance from the lingual nerve to the lingual plate of the third molar was 2.55 ± 0.55 mm. Our findings were not consistent with Kiesselbach and Chamberlein who observed the distance from the lingual nerve to the lingual crest and the lingual plate of the third molar was 2.28 and 0.58 mm respectively [3] and study by Mendes et al., where the distance from the lingual nerve to the alveolar crest and the lingual plate of the third molar was found to be 16.8 mm and 4.4 mm respectively [18]. In the present study, the depth of the lingual nerve from the gonion was measured. The lingual nerve in the third molar area was at the depth of 24.75 ± 2.38 mm from the gonion at an angle of 500 with the base of the mandible (Table 4). This information could be useful to avoid injury to the lingual nerve while manipulating the mandibular fractures. Measurements between the right and left sides were statistically insignificant indicating the bilateral symmetry of the nerve.

To achieve a successful and uncomplicated inferior alveolar nerve block, a thorough understanding of the location of the mandibular foramen (MF) is required. The placement of local anaesthetic material extremely close to the nerve before it reaches the mandibular foramen is linked to a satisfactory inferior alveolar nerve block. The location of the MF is widely varied, according to data gathered from dry mandibles and radiographic images [19-21]. The location of the MF in respect to the angle of the mandible (gonion), mandibular notch, the base of the mandible, and the occlusal plane was studied in each half of the mandibles gathered from 16 cadavers in this current study. In 87.5% of the samples, the mandibular foramen was at the level of the occlusal surface of the third molar, in 6.25% of the samples, it was approximately 9.51 mm above the occlusal surface of the third molar and in the remaining samples, it was 8.19 mm below the occlusal surface of the third molar. In the studies done by Augier M et al., and Basmajian et al., the position of the MF was described to be slightly above the level of the occlusal plane [22,23], whereas Thangavelu et al., illustrated that the location of MF lies either at or below the level of the occlusal plane [24]. Despite the fact that a few studies have documented the MF in regard to the angle of the mandible and ramus of the mandible [20,25], the angle of the mandible and the angle created by the mandibular foramen with the base of the mandible were the focus of our investigation. The mandibular foramen was located 21 ± 3.33 mm superior to the gonion at an angle of 98° ± 5° with the base of the mandible. The relationship between the MF and mandibular notch was probed and the foramen was found to be located 20.13 ± 3.1 mm inferior to the lowest point of the mandibular notch. This is similar to the findings of Oguz and Bozkir, who demonstrated that the shortest distance between the mandibular foramen and the mandibular notch was 22.27 mm [26]. Measurements between the right and left sides were statistically insignificant, indicating the bilateral symmetry of the MF, in turn the inferior alveolar nerve which is similar to the findings of Tsai HH [27] and Braga et al [28].

If the accessory mandibular foramen (accessory MF) is present, the inferior alveolar nerve block may be unsuccessful. They may convey the branches of the inferior alveolar nerve and arteries, resulting in the failure of many dental treatments to accomplish inferior alveolar nerve block [29]. In our research, accessory MF was identified inferior to the mandibular foramen in 9.37% of the samples. Accessory MFs were found below the mandibular foramen in 22.07 percent of the mandibles and above the mandibular foramen in 25.22% of the population in a Brazilian research [30]. Knowing if the accessory MF is present might help to avoid neurovascular problems during treatments targeting the ramus of the mandible.

## Conclusions

Masseteric nerve, lingual nerve, and inferior alveolar nerve have got wide applications in various dental procedures and surgical manoeuvres. Iatrogenic injuries and the inability to establish adequate local anaesthetic are frequently caused by a lack of proper topographical bony landmarks for the identification of the branches of these nerves. In this study, the topography of the masseteric nerve, lingual nerve, and inferior alveolar nerve was studied using constant and reliable bony landmarks in the cadaver which might aid effective dental and facio-maxillary surgical procedures. Since the bony landmarks are fixed and reliable, they could be used effectively in clinical practice. However, the outcome of this study could not be applied to paediatric patients as the subjects were restricted to adult cadavers. Similar sex differences were also not taken into consideration. The cadaveric study when combined with clinical study (radiological study) will be more effective than an isolated cadaveric study.
